# Boosting health care reform and servicing people health - project description of national digital health key technology and application of regional model

**DOI:** 10.1186/1479-5876-10-S2-A53

**Published:** 2012-10-17

**Authors:** Lanjuan Li

**Affiliations:** 1 The First Affiliated Hospital of Medical School of Zhejiang University, Hangzhou, 310009, China

## 

"Eleventh Five-Year Plan” National Science and Technology Support Key Project--National Digital Health Key Technology and Application of Regional model ": By constructing uniform standard Electronic Health Records (EHR), Electronic Medical Records (EMR), Interactive Health Care Information Platform, Two-way Referral of the communities and hospitals, telemedicine, distance education and health consultation system; By sharing digitization of health care resource, digitization of medical services, digitization of urban and rural community health services, digitization of public health services and protection regional model to effectively upgrade the disease prevention and control, capabilities of public health emergency responses, to improve service availability, to promote the reform and development of medical and health system, so as to achieve breakthroughs in information silos, integrate health care resource, optimize service processes, improve the medical treatment efficiency, reduce medical costs, make better relations with doctor-patient, protect people's health, achieve the goal of “Everyone has basic medical and health services”. The implementation of National Digital Health project get a high opinion from leaders at all levels and domestic and foreign experts, its research get a tremendous impact on construction of health information, play a technical supporting role of boosting health care reform and servicing people health, and make important significance to promote the development of Chinese public health care.

